# Differing metabolic responses of guard cells to blue light

**DOI:** 10.1111/nph.70375

**Published:** 2025-07-04

**Authors:** Alisdair R. Fernie, Stefan Timm

**Affiliations:** ^1^ Max Planck Institute of Molecular Plant Physiology Am Mühlenberg 1 Potsdam‐Golm 14476 Germany; ^2^ Plant Physiology Department University of Rostock Albert‐Einstein‐Straße Rostock D‐18059 Germany

**Keywords:** blue light, guard cells, metabolic responses, starch degradation, stomata

## Abstract

This article is a Commentary on Bahadar *et al*. (2025), **248**: 2347–2360.

Plants have adjustable pores, stomata, surrounded by a pair of guard cells. Alterations in the metabolism of these guard cells govern the opening and closure of the stomata in response to both endogenous and environmental signals (Sussmilch *et al*., 2019). Stomatal opening facilitates the influx of CO_2_ for photosynthesis alongside the transpiratory efflux of water, as they balance optimal growth against dehydration during drought periods. The regulation of stomatal movement is highly complex and subject to a number of triggers, including phytohormones and metabolite levels of both the guard cells themselves and other surrounding cell types (Flütsch *et al*., 2020). One prominent suggestion in the literature, principally derived from work in Arabidopsis, is that starch degradation is of particular importance during exposure to blue light (Daloso *et al*., 2017). However, considerable evidence has also been accrued supporting roles for both K^+^ and malate in guard cell osmoregulation (Outlaw & Lowry, 1977; Medeiros *et al*., 2018). Moreover, while the importance of guard cell starch degradation in stomatal opening is supported by several convincing lines of evidence, several other studies revealed that guard cell starch content was not correlated with aperture under either blue or white light (Heath, 1947; Daloso *et al*., 2017). In a study published in this issue of *New Phytologist*, Bahadar *et al*. (2025; pp. 2347–2360) performed a detailed metabolic and stomatal analysis following the exposure of cowpea and tobacco to blue light, with results suggesting that changes in both starch degradation and downstream aspects of primary metabolism are species‐specific and thus urging caution on making generalized statements concerning guard cell metabolism (Fig. 1).

*… blue light‐induced stomatal opening does not involve starch remobilization in Arabidopsis, cowpea or tobacco guard cells but rather that the level of starch ranges according to the experimental condition*.

**Fig. 1:**
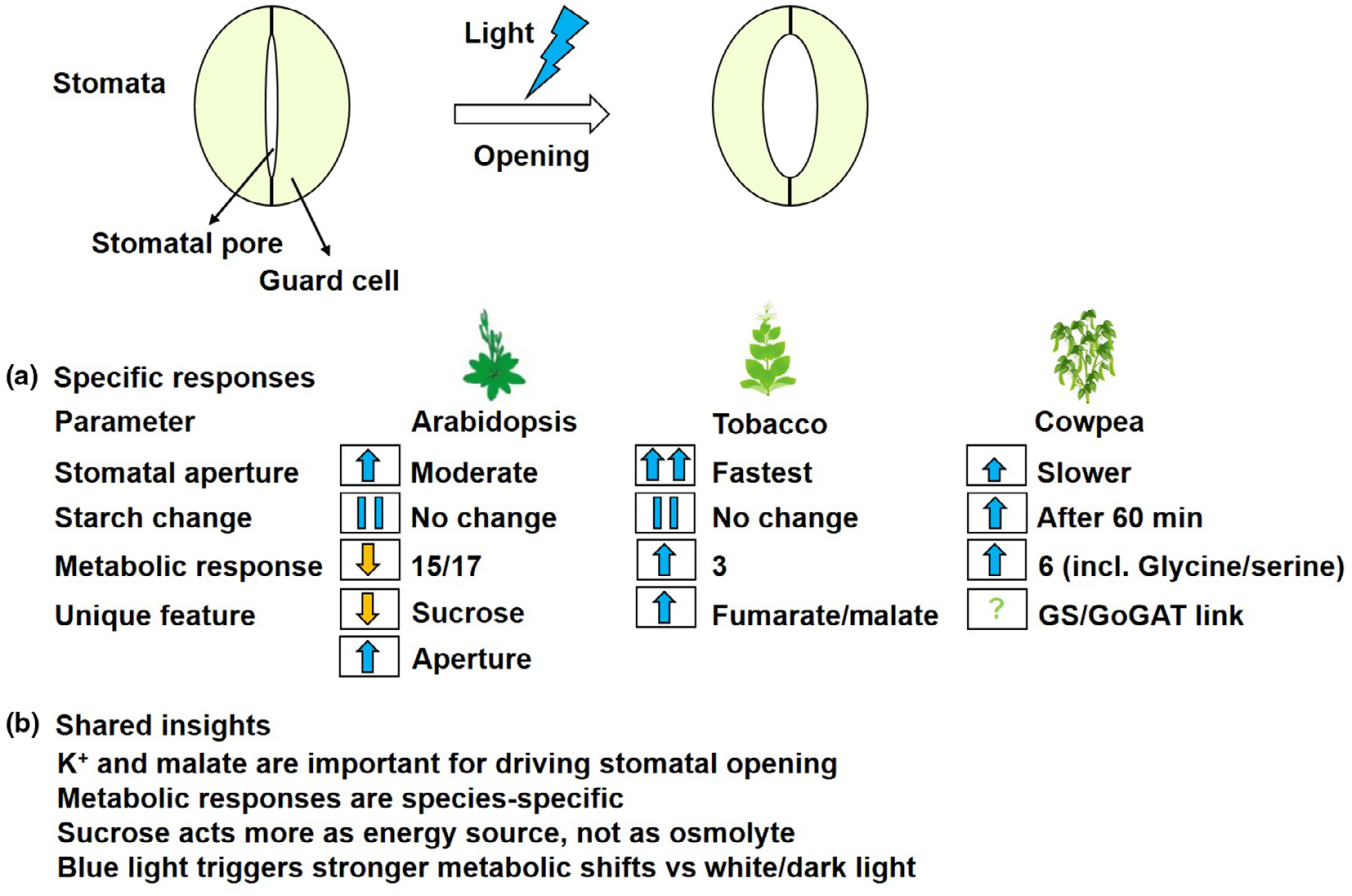
Species‐specific metabolic responses of guard cells to blue light. Stomata open in response to blue light exposure. It is hypothesized that starch degradation is involved in facilitating stomatal opening, as several previous results suggested a positive correlation of starch degradation with stomatal aperture. However, the recent study by Bahadar *et al*. (2025; **248**: 2347–2360, in this issue of *New Phytologist*) revealed that blue light‐induced stomatal opening is rather a multimetabolic, species‐dependent process, not uniformly dependent on starch. Hence, the obtained results point to the necessity for further exploration into metabolite‐specific control mechanisms.

As an initial experiment, Bahadar *et al*. tested the responsiveness of cowpea and tobacco stomata to blue light, finding that stomatal aperture increased linearly for 30 min. Given that contradictory results are presented in the literature for these species, Bahadar *et al*. carried out a more detailed analysis of the kinetics of stomatal conductance revealing that both species are indeed responsive to blue light but that both the magnitude and the speed of the increase were higher in tobacco than in cowpea (Fig. 1). However, in contrast to previous reports (Flütsch *et al*., 2020), neither Arabidopsis nor tobacco guard cells exhibited altered starch content in their guard cells, but cowpea displayed elevated starch content after 60 min of blue light treatment, prompting Bahadar *et al*. to carry out a range of experiments to address the reason behind these discrepancies. In doing so, they were able to discard the possibility that the differences between their results and those previously reported were the consequence of their experimental procedure or guard cell‐enriched epidermal fragments. Leading them to conclude that blue light‐induced stomatal opening does not involve starch remobilization in Arabidopsis, cowpea or tobacco guard cells but rather that the level of starch ranges according to the experimental condition.

In order to better understand the response to blue light, Bahadar *et al*. next evaluated changes in primary metabolite levels following exposure to blue light using a well‐established gas chromatography mass spectrometry approach. For this purpose, guard cells were extracted predawn, pooled in a hypertonic solution and washed extensively before transfer to blue light. These studies revealed considerable changes in metabolite levels in all three species, with the levels of 15 of the 17 metabolites identified in Arabidopsis decreasing after 60 min of exposure to blue light. By contrast, only three metabolites increased in tobacco during blue light treatment and six metabolites increased in cowpea including the photorespiratory metabolites glycine and serine – a fact that is perhaps pertinent given the recent demonstration of the importance of photorespiratory metabolism in guard cells (Sun *et al*., 2025). To better gauge which metabolites are associated with the dynamics of blue light‐induced stomatal opening, Bahadar *et al*. normalized their data in order to carry out a K‐means clustering analysis. For tobacco, no clear patterns between changes in metabolite levels and stomatal aperture were apparent. By contrast, stomatal aperture parameters clustered with aspartate, maltotriose, pyroglutamate, serine and urea in cowpea – with the data also suggesting that sucrose and glutamate are degraded while glucose, maltotriose and pyroglutamate are synthesized during blue light‐induced stomatal opening in cowpea. Moreover, statistical analyses of the data revealed that tobacco exhibited the greatest metabolic response to blue light, followed by cowpea and then tobacco. Collectively, the data suggest that the dynamic of blue light‐induced stomatal opening is associated with diverse metabolic changes in Arabidopsis, tobacco and cowpea. Finally, in order to understand how specific these responses are to blue light, the authors compared their data with that recently obtained for tobacco guard cells exposed to either darkness or white light (Lima *et al*., 2023). This comparison revealed that the response of metabolism to blue light is quite distinctive, with all metabolites, with the exception of fumarate, displaying higher levels under blue light than white light in tobacco. Notably, both fumarate and malate were higher in both blue light and white light than they were in darkness.

These data collectively provide a more complete picture concerning the stomatal opening properties of blue light, which have now been researched for over 50 years (Zeiger & Hepler, 1977). That said, although blue light responses have been observed in the basal lineages of plants, certain experiments in certain species were not able to identify blue light stomatal responses (Vialet‐Chabrand *et al*., 2021). The species examined in the study of Bahadar *et al*. have all been previously documented to exhibit stomatal responses to blue light. In their study, Bahadar *et al*. also demonstrated responsiveness, but increases in stomatal conductance and speed of response were considerably greater in tobacco than in cowpea (Fig. 1). The kinetic analyses of the responses revealed that they preceded starch degradation under the conditions of the study; thus, while a great deal of evidence has accrued indicating that starch metabolism of guard cells is involved in stomatal closure (Azoulay‐Shemer *et al*., 2016; Flütsch *et al*., 2020), starch‐derived metabolites do not appear to be the major contributors to blue light‐induced initial stomatal opening. As the authors correctly underlined, these data do not imply that starch degradation is unimportant in the regulation of blue light‐induced opening, and it is indeed likely important to keep the stomata open following the rapid increase in stomatal opening mediated by the accumulation of K^+^ and its counterions alongside the degradation of storage reserves (McLachlan *et al*., 2016; Medeiros *et al*., 2018; Lima *et al*., 2023). In keeping with such a model, it is important to note that sucrose levels were negatively correlated with stomatal aperture in both Arabidopsis and cowpea, in agreement with the role of sucrose in stomatal movement being purely energetic (Fig. 1). Intriguingly, while the stomatal responses were broadly similar, the changes in metabolite levels were highly diverse across the species studied. That said, a common connection among sugars and tricarboxylic acids was observed, as was a clear alteration in glutamine levels. The latter led to the authors proposing the intriguing hypothesis that the glutamine synthetase/ glutamate synthetase cycle may aid in optimizing stomatal opening. While this hypothesis will need considerable further experimental work to validate the data presented, the study of Bahadar *et al*. represents a significant milestone in dissecting metabolic aspects of the blue light response of guard cells, which will undoubtedly form a solid foundation for such future studies.

## Disclaimer

The New Phytologist Foundation remains neutral with regard to jurisdictional claims in maps and in any institutional affiliations.
